# Unraveling the Causal Relationship Between Non-Communicable Diseases, Obesity, and Health Expenditure: Insights from the Toda–Yamamoto Approach

**DOI:** 10.3390/healthcare13010001

**Published:** 2024-12-24

**Authors:** Salim Yılmaz, Canser Boz, Furkan Alp Eren, Ahmet Murat Günal

**Affiliations:** 1 Department of Health Management, Faculty of Health Sciences, Acibadem Mehmet Ali Aydinlar University, 34752 Istanbul, Turkey; 2 Department of Health Management, Faculty of Health Sciences, Istanbul University-Cerrahpaşa, 34320 Istanbul, Turkey; canser.boz@iuc.edu.tr; 3 Department of Family Medicine, Faculty of Medicine, Istanbul Medeniyet University, 34700 Istanbul, Turkey; drfurkanalperen@gmail.com; 4 Faculty of Health Sciences, Department of Nutrition and Dietetics, Haliç University, 34060 Istanbul, Turkey; ahmetmuratgunal@halic.edu.tr

**Keywords:** obesity-driven healthcare costs, NCD prevalence dynamics, causal health expenditure analysis

## Abstract

Background/Objectives: Understanding the relationship between non-communicable diseases (NCDs), obesity, and health expenditure is crucial for developing effective public health policies, particularly in light of the rising global burden of NCDs and obesity. Therefore, this study aimed to investigate the causal relationships between NCDs, obesity, and health expenditure in Turkiye. Methods: Data were collected from the World Health Organization and Our World in Data. Time series econometric analysis was performed using the Toda–Yamamoto causality approach. A model was designed to regularly capture causal relationships to ensure robust and consistent findings. Results: The analysis revealed four significant results. First, a causal relationship was observed between obesity and the prevalence of NCDs, indicating that higher obesity rates lead to an increase in NCDs within the population. Second, obesity had a direct impact on health expenditures, as rising obesity levels drove up healthcare costs. Third, the burden of NCDs contributed to increased health expenditure. Finally, the combined effect of obesity and NCDs on health expenditure was statistically significant at the 0.05 level. Conclusions: These results highlight the need for policymakers to develop more effective strategies to address both obesity and NCDs. Recommended policies include the implementation of public health programs aimed at preventing obesity, strengthening early diagnosis and treatment methods, and increasing awareness campaigns focused on NCDs. These measures would be crucial steps in improving public health and controlling healthcare expenditures.

## 1. Introduction

Non-communicable diseases (NCDs) represent one of the foremost health challenges of the 21st century. According to the World Health Organization (WHO), NCDs, or chronic diseases, are long-lasting conditions that are influenced by genetic, physiological, environmental, and behavioral factors [1]. The main types of NCDs include cardiovascular diseases, cancers, chronic respiratory conditions, diabetes, and mental health disorders. Conditions such as hyperlipidemia, metabolic disorders, obesity, neurological issues, and musculoskeletal diseases are also categorized under NCDs.

Recently, factors such as shifts in lifestyle, environmental changes, aging populations, evolving dietary patterns, and increased life expectancy have led to an increase in NCDs, such as cancer, diabetes, chronic respiratory diseases, and coronary heart disease, which are major causes of death and disability worldwide [2]. NCDs account for 41 million deaths annually, accounting for three-quarters of global mortality rates [1] A significant portion of these deaths, referred to as “premature mortality”, occur in individuals aged 30–70 years. NCDs account for approximately 65.5% of all deaths globally and are responsible for approximately 87% of deaths in individuals aged 30–70 years in Turkey [3,4].

Low- and middle-income countries bear a disproportionately high share of these deaths [5]. Alwan noted that cardiovascular diseases alone claim three times as many lives in developing countries as HIV, tuberculosis, and malaria combined, with even higher numbers in developed nations [2].

NCDs hold a prominent position on the global health agenda due to their profound impact and potential for prevention. The encouraging fact is that most of these diseases are largely “preventable” despite their significant influence on global health. Prevention efforts center around straightforward, cost-effective strategies, such as promoting healthy diets, encouraging regular physical activity, reducing alcohol consumption, and smoking cessation. Their primary aim is to mitigate the key risk factors associated with NCDs, particularly poor dietary habits and obesity [6]

The WHO identifies obesity as a growing global epidemic that significantly affects individuals’ mental, social, and physical well-being [7]. The scale of this epidemic has substantial social and economic consequences. Obesity, which is often driven by unhealthy diets and sedentary lifestyles, has far-reaching impacts that extend beyond individual health [8]. According to WHO data, the global prevalence of obesity nearly tripled between 1975 and 2016, with 13% of adults classified as obese in 2016 [9]. In Turkey, obesity rates increased from 7.6% in 1975 to 32.2% in 2016 [10], and in 2019, the country recorded 95.8 obesity-related deaths per 100,000 people [6].

Although obesity was once linked to economic affluence, it now presents a significant burden for both developed and developing nations, leading to substantial healthcare costs. he global economic impact of obesity was nearly USD 2 trillion in 2020 and is projected to exceed USD 3 trillion by 2030 and USD 18 trillion by 2060 [11]. In Turkey, obesity’s financial toll is estimated to be TRY 186 billion annually [12]. In the US, obesity-related illnesses account for 5.5% of all health expenditures [13].

Countries’ health systems face a dual challenge of managing both communicable diseases and NCDs, with NCDs emerging as a growing threat to public health and economic development. Recently, concerns about the rise of NCDs have intensified, prompting many nations to implement targeted action plans within their public health strategies to curb their prevalence. However, addressing NCDs effectively requires national initiatives and global cooperation. The United Nations held High-Level Meetings on the Prevention and Control of Non-Communicable Diseases in 2011, 2014, and 2018 to engage heads of state and foster political commitment to combating NCDs.

The spread of NCDs has significant human, social, and economic consequences. These diseases reduce economic productivity by negatively impacting the size and quality of the labor force, ultimately leading to income and output losses [14]. Additionally, NCDs increase health expenditures, particularly because they often require long-term, ongoing treatment and care. Bloom et al. studied the economic burden of NCDs in China and India and reported that the five major NCDs cost China USD 27.8 trillion and India USD 6.2 trillion according to the EPIC model [15]. Similarly, Vandenberghe and Albrecht conducted a systematic review in the European Union, which showed that NCDs significantly increase healthcare costs [16]. Additionally, Muka et al. reported a positive link between rising NCD prevalence, rising health expenditures, and declining societal well-being [17]. A previous study in Germany highlighted the macroeconomic impact of cardiovascular diseases on health spending since 2003 [18].

This study aimed to investigate the causal relationships between NCDs, obesity, and health expenditures, with a particular focus on their combined effects. While the individual impacts of obesity and NCDs on health systems are well documented, the combined burden of these two factors has received limited attention in the existing literature. This study not only examines whether obesity drives the prevalence of NCDs and inflates healthcare costs but also explores whether their combined effects amplify health expenditures beyond their individual contributions. By utilizing macrolevel data rather than clinical data, this study provides a broader perspective on population health trends and aims to identify actionable strategies to mitigate the combined burden of obesity and NCDs on healthcare systems.

## 2. Materials and Methods

A model capable of detecting strong causal relationships between obesity, NCD prevalence, and health expenditure across consistent and successive time periods was employed to ensure the accuracy and reliability of the findings. In this study, econometric causality models were employed to analyze the relationships between variables. The burden of NCDs was measured annually using disability-adjusted life years (DALYs), a metric that captures the total impact of disease by considering both premature mortality and years lived with disability. One DALY represents the loss of one year of a healthy life. Epidemiologists categorize disease burden into three main groups: NCDs [blue], communicable, maternal, neonatal, and nutritional diseases [red], and injuries [green] [19]. Data were obtained from Our World in Data. DALYs have been measured in the Global Burden of Disease (GBD) study by the Institute of Health Metrics and Evaluation (IHME) since 1990, and by the “Disease Burden Unit”, which was created in 1998 at the World Health Organization (WHO). They were also prominently featured in the World Bank’s 1993 World Development Report. Moreover, the data in Our World in Data are gathered from The Global Burden of Disease (GBD) study, which provides a comprehensive assessment of global health trends. This dataset contains the death and DALY counts and rates for 371 diseases and injuries.

**Figure 1:**
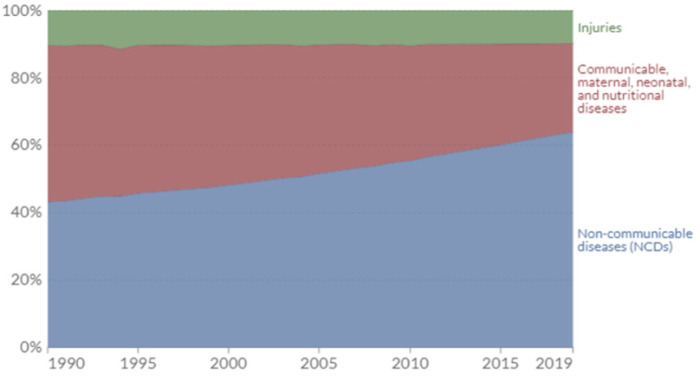
Global disease burden by cause between 1990 and 2019.

In 2019, over 60% of the global disease burden was attributed to NCDs, with communicable, maternal, neonatal, and nutritional diseases accounting for 26.35%. Injuries accounted for less than 10% of the total burden (Figure 1).

Obesity was measured using the Body Mass Index (BMI). The WHO defines BMI as “a simple index of weight-for-height that is often used to classify individuals as underweight, normal weight, overweight, or obese”. BMI values are used to determine an individual’s weight category, with the WHO setting specific thresholds: a BMI between 25.0 and 30.0 kg/m^2^ is categorized as “overweight”, whereas a BMI ≥ 30.0 kg/m^2^ is categorized as “obese”. In this study, the percentage of individuals with a BMI of ≥30 was used to represent obesity within the population. Data for this indicator were obtained from the WHO.

Current health expenditure was used as a percentage of gross domestic product (GDP) to measure healthcare costs. This indicator reflects total spending on healthcare goods and services within a given year, excluding capital expenditures, such as infrastructure, equipment, information technology, and vaccine reserves for emergencies or outbreaks. Data for this measure were obtained from the World Bank.

Annual data from 1990 to 2019 were analyzed to examine the relationships between obesity, NCDs, and health expenditure. Causality was estimated using E-Views 9.0 econometric software. A detailed summary of the variables is shown in Table 1.

The Toda–Yamamoto causality approach was employed, which is a widely favored method for obtaining robust results when investigating dual causality relationships between variables [20]. The foundational theory of causality was first introduced by Clive W. Granger in 1969 in his seminal paper, “Investigating Causal Relations by Econometric Models and Cross-Spectral Methods” [21]. According to Granger’s theory, causality between two variables can be examined by assessing whether the lagged values of one variable contribute to predicting the current value of the other. Particularly, if the inclusion of past values of variable X enhances the explanatory power of a model for variable Y at time t, X is said to cause Y.

In 1980, Sims made a significant contribution to this approach by extending the theoretical and empirical framework of Granger causality [22]. Since then, various causality tests have been developed, including the Toda–Yamamoto Causality Test and Panel Causality Test, which were built on the work of Granger and Sims [23]. One key distinction of the Toda–Yamamoto method is that it does not require the series to be stationary, unlike Granger’s original approach, which necessitates the stabilization of the series [21]. Instead, the Toda–Yamamoto method allows the inclusion of level values in the analysis, even when the variables are not stationary.

Toda and Yamamoto demonstrated that the modified Wald test can be applied effectively by estimating the level values of variables, even without stationarity [20]. This method is particularly useful when the maximum order of integration (dmax) is smaller than the lag length (k) in the causality analysis. By adding an extra lag corresponding to the maximum integration level, the model is estimated as [k + (dmax)], and the modified Wald hypothesis test is performed. This approach ensures that the time series data retain more information, which results in more accurate and reliable results [20,24,25].

In this study, the Toda–Yamamoto econometric method was applied to explore potential causal relationships between the variables. Particularly, whether bidirectional causality existed was examined, and the research hypotheses were formulated accordingly:

- H_0a_: Obesity is not the cause of the burden of NCDs.
- H_1a_: Obesity is the cause of the burden of NCDs.
- H_0b_: Obesity is not the cause of health expenditures.
- H_1b_: Obesity is the cause of health expenditures.
- H_0c_: The burden of NCDs is not the cause of health expenditures.
- H_1c_: The burden of NCDs is the cause of health expenditures.
- H_0d_: The interaction between NCDs and obesity is not the cause health of expenditures.
- H_1d_: The interaction between NCDs and obesity is the cause of health expenditures.

According to the Toda–Yamamoto causality approach, the variables can be elucidated through three distinct equations:

$${NCD}_{t}=\beta_{0a}+\sum_{i=1}^{k}\left(\alpha_{1i}{NCD}_{t-i}\right)+\sum_{j=k+1}^{dmax}\left(\beta_{2j}{NCD}_{t-j}\right)+\sum_{i=1}^{k}\left(\alpha_{1i}{OBE}_{t-1}\right)+\sum_{j=k+1}^{dmax}\left(\theta_{2j}{OBE}_{t-i}\right)+\varepsilon_{1t}\;for\;\left[1-Hypotheses\;\alpha \right]\;{HE}_{t}=\beta_{0a}+\sum_{i=1}^{k}\left(\alpha_{1i}{HE}_{t-i}\right)+\sum_{j=k+1}^{dmax}\left(\beta_{2j}{HE}_{t-j}\right)+\sum_{i=1}^{k}\left(\alpha_{1i}{OBE}_{t-1}\right)+\sum_{j=k+1}^{dmax}\left(\theta_{2j}{OBE}_{t-i}\right)+\varepsilon_{2t}\;for\;\left[2-Hypotheses\;\alpha \right]\;{NCD}_{t}=\beta_{0b}+\sum_{i=1}^{k}\left(\alpha_{1i}{HE}_{t-i}\right)+\sum_{j=k+1}^{dmax}\left(\beta_{2j}{HE}_{t-j}\right)+\sum_{i=1}^{k}\left(\alpha_{1i}{NCD}_{t-1}\right)+\sum_{j=k+1}^{dmax}\left(\theta_{2j}{NCD}_{t-i}\right)+\varepsilon_{3t}\;for\;\left[3-Hypotheses\;\alpha \right]\;{HE}_{t}=\beta_{0a}+\sum_{i=1}^{k}\left(\alpha_{1i}{HE}_{t-i}\right)+\sum_{j=k+1}^{dmax}\left(\beta_{2j}{HE}_{t-j}\right)+\sum_{i=1}^{k}\left(\gamma_{1i}[{{NCD}_{t-1}*OBE}_{t-1}]\right)+\sum_{j=k+1}^{dmax}\left(\delta_{2j}[{NCD}_{t-i}*{OBE}_{t-i}]\right)++\varepsilon_{2t}\;for\;\left[4-Hypotheses\;\alpha \right]$$

The unit root test was performed to determine whether the series was stationary. This test helped identify the degree of integration (dmax) of the series, which is a critical step for determining the number of additional lags required in the model according to the Toda–Yamamoto approach. Next, the Toda–Yamamoto method was used, which allowed for estimation regardless of whether the series was stationary. Thanks to this approach, causality between the series has been uncovered reliably, and the hypothesis has been tested by the modified Wald test. By addressing the issue of nonstationarity, accurate results were obtained, and the findings were interpreted at a 95% confidence level.

## 3. Results

The Augmented Dickey–Fuller (ADF) unit root test was performed to determine the maximum degree of integration (dmax) of the variables. Table 2 shows the results of the ADF test.

Based on the results of the ADF unit root test, the health expenditure and NCD series were integrated of order 1 (I(1), stationary after first differencing), whereas the obesity series were integrated of order 2 (I(2)). Therefore, the maximum degree of integration (dmax) was determined as 1 for the health expenditure and NCD causality analysis and 2 for the obesity and NCD and the obesity and health expenditure causality analyses.

Subsequently, the maximum integration degree for each series in the model was determined before performing the optimal lag length selection and causality analysis. The optimal lag length (k) of the equations was calculated using the method developed by Schwert [26]. Information criteria, including the Akaike information criterion, Schwarz criterion, and Hannan–Quinn information criterion, were employed to select the optimal lag lengths. According to these criteria, the optimal lag length was determined to be one for the first two models and two for the third model. Under these conditions, the sum of k and dmax was determined to be 3 for all equations. The causality test results, based on the models with k + dmax, are shown in Table 3, which outlines the results of the modified Wald test.

**Figure 2:**
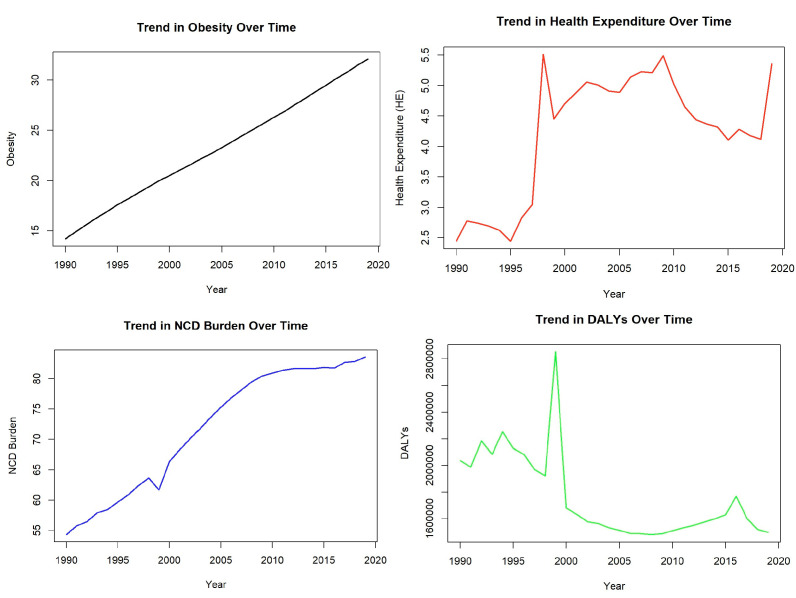
Trends in indicators in Türkiye (1990–2019).

Between 1990 and 2020, obesity rates exhibited a consistent upward trend, doubling from approximately 15% to over 30%, indicating a growing public health challenge. Concurrently, health expenditure (% of GDP) showed a sharp increase during the mid-1990s, followed by fluctuations in the 2000s, and a pronounced rise approaching 2020. The burden of non-communicable diseases (NCDs), as measured by DALYs, also demonstrated a steady increase, particularly from 1990 to 2005, with a slower upward trajectory thereafter. Notably, DALY trends revealed a sharp peak around 2000, followed by a significant decline and stabilization, before showing a slight rise post-2015. These trends collectively underscore the compounding impact of obesity and NCDs on healthcare systems, emphasizing the critical need for targeted public health interventions to address their combined burden and mitigate rising health expenditures (Figure 2).

The analysis revealed that the initial null hypothesis, which posited that obesity is not a driving factor behind the disease burden, was rejected. Similarly, the second null hypothesis, stating that obesity does not significantly contribute to health expenditures, was rejected. Moreover, the third null hypothesis, that the burden of NCDs is not the cause of health expenditures, was also rejected. Consequently, the results of the causal models confirmed causal relationships between obesity, the burden of NCDs, and health expenditures. Furthermore, the disease burden, measured by DALYs, contributed to healthcare spending. Additionally, the null hypothesis for the interaction between obesity and NCDs on health expenditure could not be rejected (χ^2^ = 2.085, df = 4, *p* = 0.104), although the result was close to the 10% significance threshold, indicating that the combined effect of obesity and NCDs does not significantly drive healthcare costs.

## 4. Discussion

This study investigated the causal relationships between obesity, NCDs, and health expenditure in Türkiye from 1990 to 2019 using the Toda–Yamamoto causality approach. Our findings establish a robust causal relationship between obesity and the prevalence of NCDs, highlighting its critical role as a driver of chronic disease burden. The observed association aligns with prior research demonstrating the significant impact of obesity on NCD risk. A study analyzing data from 195 countries between 1990 and 2017 states that high BMI was linked to 4.7 million deaths and 147.7 million DALYs from NCDs globally, with the leading causes being ischemic heart disease, stroke, and diabetes mellitus in 2017. Projections rising to 5.5 million deaths and 176.9 million DALYs by 2025 underscore the increasing global burden of high BMI [27]. A systematic analysis of the Global Burden of Disease Study 2019 further affirms these findings by stating that ischemic heart disease and stroke were the top-ranked causes of DALYs in the 50-year-old and older age groups in 2019 and, since 1990, there has been a marked shift toward a greater proportion of burden due to years lived with disability (YLDs) from non-communicable diseases [28]. Similarly, studies conducted in Türkiye report that nearly half of type 2 diabetes cases were associated with obesity [29], and 80.9% of patients with a history of cardiovascular events were overweight, with 30% being obese [30]. Additionally, an umbrella review of meta-analyses confirms that obesity was related to the increased incidence of certain types of cancer [31]. This relationship may be attributed to unhealthy dietary habits [32]; the pro-inflammatory state and metabolic dysregulation caused by excess adiposity may foster the onset and progression of these conditions [33]. A 10-year projection study conducted in Brazil reports that if the current prevalence of overweight is maintained, the incidence of NCD cases and deaths could be reduced by 3.3% and 1.5%, respectively, compared to the continuation of current trends [34]. The findings reinforce the urgency of addressing obesity as a modifiable risk factor for reducing NCD prevalence.

Our results indicate a direct causal relationship between rising obesity levels and increased healthcare expenditures, consistent with prior economic evaluations [11,35]. Obesity contributes to healthcare costs by associations with higher rates of hospitalization, medical procedures, and long-term medication use. A study conducted in Ghana reported that compared with normal-weight participants, obesity was associated with 159% more inpatient admissions, and with 53% additional outpatient visits. The average per person total cost for obesity was USD 132 compared, with USD 35 for normal weight [36]. A study investigating the causal effect of obesity on direct medical care costs in the United States (US) stated that adults with obesity compared with those with normal weight experienced higher annual medical care costs by 100%, with costs increasing significantly with class of obesity, from 68.4% for class 1 to 233.6% for class 3 [37]. Another study on the current and estimated economic impacts of overweight and obesity including 161 countries states that the cost is estimated at 2.19% of GDP in 2019, and if current trends continue, it is projected to rise to 3.29% of GDP globally by 2060 [38]. Wang et al. [39] used a simulation model to project the probable health and economic consequences of a continued rise in obesity in the US and the United Kingdom (UK). They estimate that there will be 65 million more adults with obesity in the US and 11 million more in the UK by 2030, and the combined medical costs associated with the treatment of obesity-related diseases will increase by USD 48–66 billion in the US and by GBP 1.9–2 billion per year in the UK by the same year. These insights underscore obesity’s dual impact on public health and healthcare expenditure, warranting comprehensive strategies to mitigate its effects.

This study’s findings also confirm a causal link between the burden of NCDs and increased health expenditures, which aligns with global patterns reported in the literature [17,40,41]. NCDs are associated with high treatment costs, particularly for conditions requiring long-term management such as diabetes, cardiovascular diseases, and cancer. For instance, a systematic review reported that NCDs in the European Union (EU) claim at least 25% of the total healthcare budget and impose an important economic loss, with about 2% of GDP. The economic toll of NCDs in the EU is projected to increase substantially in the coming decades due to aging populations and rising prevalence rates [17].

However, our results suggest that the interaction effect between obesity and NCDs on healthcare costs was not statistically significant. This finding contrasts with previous studies that reported synergistic effects, where obesity amplified the cost burden of NCD management [16,42]. For instance, Lartey et al. [36] state that one in five of the overweight and obese population had at least one chronic disease, and having a chronic disease was associated with increased outpatient utilization. A study estimates the direct healthcare cost of overweight and obesity in South Africa, reporting that this cost can be disaggregated into several NCDs such as cancers, cardiovascular diseases, and diabetes [43]. Further research incorporating more granular data, such as age- or gender-stratified analyses, may provide deeper insights into these dynamics. While the independent effects of obesity and NCDs on health expenditures are clear, the nuances of their combined impact require further exploration to inform integrated intervention strategies.

The application of the Toda–Yamamoto causality approach represents a methodological strength of this study, as it enables robust causal inference without requiring stationarity in the data. This feature is particularly advantageous when analyzing complex, real-world datasets, where stationarity assumptions are often unmet. By accommodating the maximum order of integration, this approach ensures greater reliability in detecting causal pathways among the study variables.

However, several limitations warrant consideration. First, the reliance on macrolevel data, while useful for capturing population-wide trends, may obscure individual-level nuances, such as behavioral or genetic factors influencing obesity and NCD outcomes. Second, the causal models did not account for potential confounding variables that could affect the relationship between obesity, NCDs, and health expenditures. Third, this study’s focus on Türkiye limits the generalizability of findings to other contexts with differing demographic and healthcare profiles. Finally, this study did not fully explore the role of emerging factors, such as the influence of social media on health behaviors and the potential for technological advancements in healthcare. Future studies could address these limitations by integrating microlevel data and extending analyses to cross-national comparisons. Nevertheless, the findings highlight the need for global health policies that focus on preventing obesity and managing NCDs, especially through community-driven approaches and government-led incentivization efforts.

## 5. Conclusions

This study aimed to investigate the causal relationships between NCDs, obesity, and health expenditure in Turkiye. This manuscript revealed several key findings. First, a causal relationship was observed between obesity and the prevalence of non-communicable diseases (NCDs), indicating that higher obesity rates contribute to an increase in NCDs within the population. Second, obesity had an impact on health expenditures, as rising obesity levels led to higher healthcare costs. Third, the burden of NCDs was found to contribute to increased health expenditures. However, the causality relationship between the combined effect of obesity and NCDs on health expenditures was not statistically significant at the 5% significance level. This indicates that the interaction term between obesity and NCDs does not have a significant impact on health expenditures based on the presented data. While the proximity to the 10% significance threshold suggests a potential causality, these findings suggest that the combined effect is not as strong as the individual effects. Therefore, it is emphasized that this combined effect requires further investigation in future studies. These findings highlight the need for policymakers to develop more effective strategies to address both obesity and NCDs. Recommended policies include the implementation of public health programs aimed at preventing obesity, strengthening early diagnosis and treatment methods, and increasing awareness campaigns focused on NCDs. These measures would be crucial steps in improving public health and controlling healthcare expenditures.

## Figures and Tables

**Table 1:** Definitions of the variables.

Abbreviation	Variable Definition	Source
Obesity	Proportion of individuals with a BMI of ≥30	https://www.who.int/data/gho/data/indicators/indicator-details/GHO/prevalence-of-obesity-among-adults-bmi-=-30-(age-standardized-estimate)-(-) (accessed on 24 July 2024)
NCD	Burden of NCDs is represented by the total disease impact, measured in DALYs per year	https://ourworldindata.org/grapher/burden-of-disease-rates-from-ncds (accessed on 24 July 2024)
Health expenditure	Current health expenditure (% of GDP)	https://data.worldbank.org/indicator/SH.XPD.CHEX.GD.ZS (accessed on 24 July 2024)

**Table 2:** ADF unit root test results (intercept).

	Test Critical Values				
	*t*-Stat	%1	%5	%10	*p*-Value
Health expenditure (∆)	−6.338283 _[0]_	−3.689	−2.971	−2.625	<0.00001 *
NCD (∆)	−5.270350 * _[0]_	−3.689	−2.971	−2.625	0.0002 *
Obesity (∆∆)	−17.27366 * _[1]_	−3.699	−2.976	−2.627	0.0001 *
NCD × Obesity Interaction (∆∆)	−3.810492 * _[1]_	−3.580	−2.930	−2.600	0.03421 *

∆ = first difference; ∆∆ = second difference; [0]: No lag; [1]: 1 lag; * Statistically significant at 5%. The values in square brackets indicate the lag lengths determined by the Schwarz information criterion.

**Table 3:** Results of the causality test based on the Toda–Yamamoto method.

Hypothesis	χ^2^	df	*p*	Result
Obesity ⇏ NCD H_0_: no causality	11.82509	2	0.0005 **	H_0_: rejected
Obesity ⇏ health expenditure H_0_: no causality	16.841 07	2	<0.00001 **	H_0_: rejected
NCD ⇏ health expenditure H_0_: no causality	15.04441	2	0.00001 **	H_0_: rejected
Obesity×NCD Interaction ⇏ health expenditure H_0_: no causality	2.085039	4	0.104401	H_0_: cannot rejected

** *p*-value was calculated according to the degree of freedom.

## Data Availability

The data utilized in this study are openly accessible at the following link: https://dosya.co/znygcczkl1tv/eviews.xlsx.html (accessed on 9 November 2024).
